# AFP, PIVKAII, GP3, SCCA-1 and follisatin as surveillance biomarkers for hepatocellular cancer in non-alcoholic and alcoholic fatty liver disease

**DOI:** 10.1186/1471-2407-8-200

**Published:** 2008-07-18

**Authors:** Gary Beale, Dipankar Chattopadhyay, Joe Gray, Stephen Stewart, Mark Hudson, Christopher Day, Paolo Trerotoli, Gianluigi Giannelli, Derek Manas, Helen Reeves

**Affiliations:** 1 Paul 'O Gorman Building, Northern Institute for Cancer Research, The Medical School, Framlington Place, Newcastle University, Newcastle-upon-Tyne, UK; 2 Pinnacle Proteomics Laboratory, The Medical School, Framlington Place, Newcastle University, Newcastle-upon-Tyne, UK; 3 The Freeman Hospital Liver Unit, Freeman Road, The Medical School, Framlington Place, Newcastle University, Newcastle-upon-Tyne, UK; 4 School of Clinical Medical Sciences, The Medical School, Framlington Place, Newcastle University, Newcastle-upon-Tyne, UK; 5 Department of Biomedical Science and Human Oncology, Section of Medical Statistics, Department of Emergency and Organ Transplantation, University of Bari Medical School, Italy; 6 Department of Internal Medicine, Immunology, and Infectious Diseases, Section of Internal Medicine, University of Bari Medical School, Italy

## Abstract

**Background:**

The incidence and mortality of hepatocellular cancer (HCC) complicating alcoholic and non-alcoholic fatty liver diseases (ALD and NAFLD) is rising in western societies. Despite knowing the at risk populations for HCC development, the lack of sensitive and specific means of surveillance hampers disease detection at curable stages. The most widely used serum HCC marker is alpha-fetoprotein (AFP), while PIVKA-II, glypican-3 (GP3) and Squamous Cell Carcinoma Antigen -1 (SCCA-1) have been proposed as new biomarkers. Assessment of these HCC biomarkers has largely been performed in patients with viral hepatitis. We conducted a cross sectional study assessing the value of these serum proteins, as well a novel candidate biomarker -follistatin – in patients with HCC arising on a background of ALD or NAFLD.

**Methods:**

Pre-treatment serum samples from 50 patients with HCC arising on a background of ALD (n = 31) or NAFLD (n = 19) were assessed by specific ELISA assay for PIVKAII, Glypican-3, SCCA-1 and Follistatin. Results were compared and contrasted with a control patient group with biopsy proven steatohepatitis-related cirrhosis (n = 41). The diagnostic accuracy of each of the candidate biomarkers was evaluated using receiver operating characteristic (ROC) curve analysis, reporting the area under the curve (AUC) and its 95% confidence interval (CI). Performance was compared to that of the established biomarker, AFP.

**Results:**

Serum levels of all proteins were assessed by specific ELISA assays. GP3, SCCA-1 and follistatin had no HCC surveillance benefit in these patients. AFP and PIVKAII were superior to the other markers, particularly in combination.

**Conclusion:**

We conclude that while novel means of surveillance are urgently required, the combination of AFP and PIVKAII for HCC is an improvement on AFP alone in ALD/NAFLD patients. Furthermore, our data in this homogenous subset of patients- particularly that confirming no role for SCCA-1 – suggests that the choice of optimal biomarkers for HCC surveillance may be determined by the aetiology of underlying chronic liver disease.

## Background

Hepatocellular carcinoma (HCC) is a major health problem worldwide, with more than 500,000 cases diagnosed annually [1]. While the incidence of HCC has reportedly risen over the last 5–8 years, the survival of those affected has not changed significantly in the last two decades [1-3]. This is related to both its late detection the lack of effective therapies for advanced stage disease [4]. Up to 80% of HCCs develop against a background of cirrhosis of the liver and while we believe that surveillance of the at risk cirrhotic population could aid earlier detection of the disease and decrease the cancer related mortality rate, our present success is limited by the lack of sensitive biomarkers. Currently, standard surveillance includes a combination of 6 monthly abdominal ultrasound scan (USS) and serum alphafetoprotein (AFP) measurement, but this strategy does not reliably detect early disease. The diagnostic performance of AFP is inadequate[5] as it is only elevated in 40–60% of cases, while abdominal USS is difficult in cirrhotic nodular livers and notoriously user dependent[6]. Alternative serum biomarkers are being actively sought and proposed candidates include Prothrombin Induced by Vitamin K Absence (PIVKA-II), glypican-3 (GP3), and more recently, Squamous Cell Carcinoma Antigen -1 (SCCA-1).

PIVKA-II is an abnormal prothrombin identified as an HCC biomarker in 1984 [7] and since reported elevated most notably in advanced cases with portal vein invasion [8,9]. It is proposed that PIVKA-II may be useful primarily as a prognostic biomarker, predicting rapid tumour progression and a poorer prognosis [10]. The oncofetal antigen glypican3 (GP3) is a heparan sulfate proteoglycan that is expressed in more than 70% of HCC[11]. When combined with AFP it has a sensitivity of up to 82% for HCC detection on a background of viral hepatitis [12]. SCCA-1 is a member of the high molecular weight serine protease family called serpins [13] initially reported elevated in epithelial tumours such as the cancer of the head [14] and more recently in the serum of individuals with HCC and cirrhosis. [15]

On a global scale, viral causes of chronic liver disease are the commonest predecessors of HCC and these proposed biomarkers [16] have largely been studied in this disease group. Our own HCC patients have tumours arising predominantly on a background of alcoholic (ALD) and non-alcoholic fatty liver diseases (NAFLD). Here we present the data on a cross-sectional study comparing the efficacy of these markers, as well as a novel candidate biomarker, Follistatin, for the diagnosis of HCC arising on a background of steatohepatitis related cirrhosis. Follistatin is a secreted monomeric protein overexpressed in rat and human liver tumours and reportedly contributing to hepatocarcinogenesis by the inhibition of activins [17]. Follistatin mRNA was markedly overexpressed in HCC cell line microarray studies performed in our own laboratory (unpublished data).

Our data indicate differences in biomarker performance in NAFLD and ALD patients compared to performances reported in viral hepatitis. Neither PIVKAII, GP3, SCCA-1, nor the novel candidate Follistatin, has a role independent of AFP in HCC surveillance in steatohepatitis related cirrhosis. We show that the combination of AFP and PIVKAII is more valuable than AFP alone and suggest this approach be adopted as standard surveillance in this disease group.

## Methods

### Patient serum samples

All patient serum and clinical information were collected with patient consent after approval by The Newcastle and North Tyneside Ethics Committee approved this study. Patients were diagnosed as having HCC as per guidelines proposed by the European Association for the Study of the Liver [6]. Pre-treatment samples from 50 patients with HCC, all of whom had an underlying cirrhosis, were selected for study. Of these, 31 patients had alcoholic liver disease (ALD) and 19 patients had NAFLD. The serum was immediately separated by centrifugation and frozen at -80°C. These serum samples were compared to an independent group of 41 patients with biopsy proven ALD or NAFLD cirrhosis. The diagnosis of NAFLD cirrhosis was made in patients who had clinical features and liver biopsies compatible with NAFLD. Females and males consuming greater than 14 or 21 units of alcohol per week respectively were excluded from this category, as were any individuals with viral or autoimmune liver diseases. The presence of steatosis was necessary for the diagnosis to be made, as was stage 4 fibrosis defined by modified Brunt criteria[18]

The biochemical serum tests, including serum AFP, were measured using routine automated methods in the Biochemistry Laboratory at the Freeman Hospital, Newcastle upon Tyne. No patient positive for either HBsAg or HCV were included in this study.

### Western blotting and serum ELISA assays

PIVKA-II was measured using a commercially available ELISA kit (Asserachrom PIVKAII kit, Stago, France), according to the manufacturer's instructions. The detection limit is 01 ng/ml. The cut-off value was set as 20 ng/ml for differentiation between HCC and cirrhosis based on the findings in this study. Glypican-3 was measured using commercially available ELISA kit (Biomosaics limited) following the manufacturer's protocol. Serum SCCA-1 was measured as previously described an ELISA kit purchased from Xeptagen (Xeptagen, Naples, Italy) and following the manufacturer's instructions. [15]

Follistatin was selected for study based on its marked expression in HCC cell lines on microarray analysis and literature review identifying it as a secretory protein with a previously suspected role in hepatocarcinogenesis. Ten samples each from patients with NAFLD, NAFLD and cirrhosis, or NAFLD with cirrhosis and HCC were immunedepleted by multiple affinity removal (MARS HPLC column;Agilent technologies) and desalted using 5 K molecular weight cut off spin filters (Agilent technologies). Subsequently, 50 μg of protein was separated by SDS-PAGE and transferred to PVDF membrane (250 mA for 90 min). The membrane was then probed with mouse anti-follistatin antibody (R&D Systems) at 1:500 dilution at room temperature overnight. After washing in Tris Buffered Saline (0.1% Tween), the membranes were incubated with secondary peroxidase conjugated rabbit anit-mouse immunoglobulin and developed using ECL (Amersham). Subsequently, a direct ELISA assay for quantitative analysis, was developed using different concentrations of serum (raw; 1:10; 1:50; 1:100: 1:1000) with serial dilution of primary antibody. Optimal conditions were using a raw serum dilution of 1:10 and an antibody dilution of 1:250.

### Statistical analysis

Quantitative variables were expressed as mean and standard deviation. Comparison between groups was by Pearson Chi-square, Wilcoxon or Student's t-test, as appropriate. Qualitative variables were expressed as count and percentage and comparisons between independent groups was by Pearson Chi-square. The diagnostic accuracy of each of the candidate biomarkers was evaluated using receiver operating characteristic (ROC) curve analysis, reporting the area under the curve (AUC) and its 95% confidence interval (CI). The diagnostic cut-off and the related sensitivity and specificity were determined. Statistical analysis was performed with SAS V8.2 software for PC, MedCalc version 7.4.3.0, as well as SPPS version 14.

## Results

Serum AFP, PIVKA-II, GP3 and SCCA-1 levels were determined in 50 patients with HCC arising in a background of ALD or NAFLD cirrhosis. A control group of 41 patients with cirrhosis from ALD/NAFLD was used for comparison. The clinical characteristics of the patients in these groups are shown in Table 1.

**Table 1:** Clinical characteristics of the patients with cirrhosis and cirrhosis plus HCC

	**Cirrhosis**	**HCC**
**Number**	41	50
**Age (years)**	54.3 ± 9.62	67.5 ± 12.02
**Male:Female**	28:13	40:10
**ALD:NAFLD**	33:08	30:20
**Childs-Pugh A:B:C**	22:14:05	27:18:05
**Portal vein invasion**	NA	8
**Single nodule**	NA	22
**Two nodules**	NA	10
**≥ 3 nodules**	NA	18

The difference in age between HCC and cirrhosis alone patients was statistically significant (p < 0.001; Pearson Chi square test), while there was no significant difference between sex or Child-Pugh Stage in the two groups. NA – not applicable.

### Serum AFP and PIVKAII as biomarkers of HCC in steatohepatitis related cirrhosis

**Figure 1:**
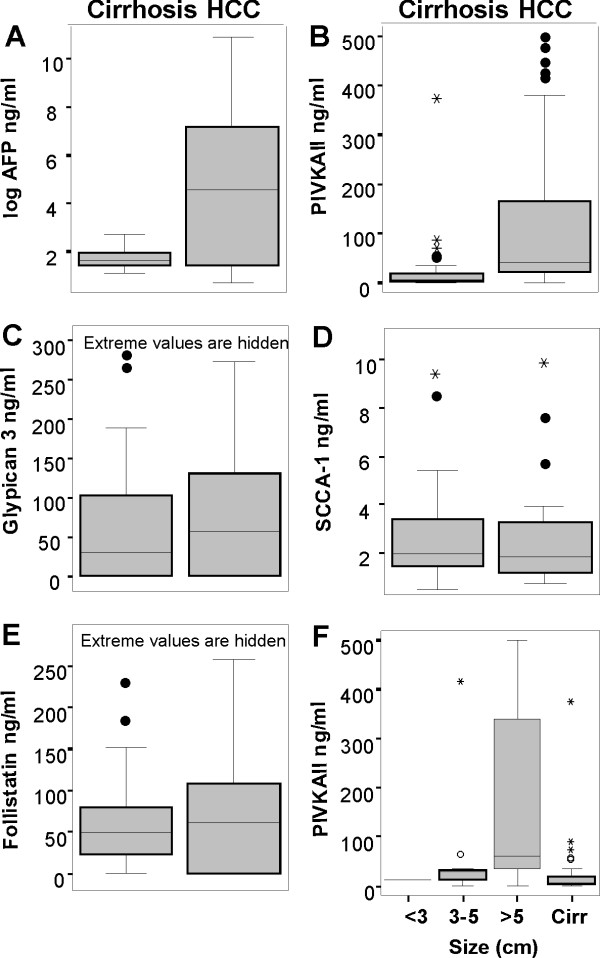
**Serum levels of Candidate Biomarkers in cirrhotic patients with and without HCC**. Box plots comparing levels of AFP, PIVKAII, GP3, SCCA-1 and Follistatin are shown. Levels are presented as ng/ml, except for AFP where the log data are presented in order to accommodate the wide range. The mean between the two groups is significantly different for both AFP and PIVKAII. For the latter, this is predominantly a result of a marked increase in levels in individuals with tumours greater than 5 cm in size.

**Figure 2:**
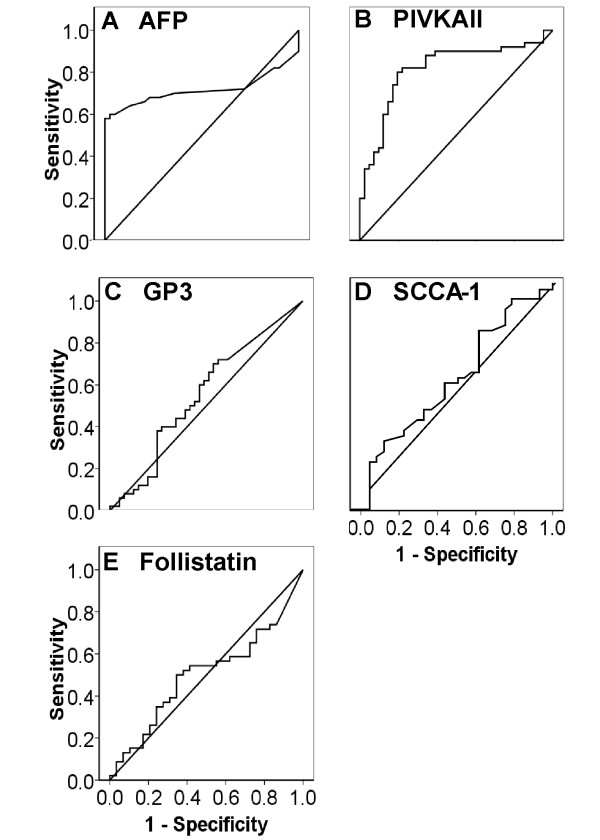
**ROC Curve analyses of the candidate biomarkers**. The diagnostic accuracy of each candidate biomarker, in terms of sensitivity and specificity, are presented after receiver operating characteristic (ROC) curve analysis. In figures 2A and 2B, corresponding to AFP and PIVKAII, the area under the curve is markedly better than for the other markers.

The median AFP value determined in our patients with HCC and those with cirrhosis were 92.4 ng/ml and 5.96 ng/ml respectively. These data are represented using a log scale in Figure 1A and also summarised in Table 2. This difference was statistically significant (p value 0.0004). The ROC curve analysis (Figure 2A) confirmed an area under curve of 0.71 (CI 95% 0.61 – 0.8), with a cut-off value of 15 giving a sensitivity of 58% (CI95% 43.2% 71.8%) and a specificity of 100% (CI 95% 91.3 – 100).

**Table 2:** Levels of candidate biomarkers (ng/ml) as detected by specific ELISA assays

	**Cirrhosis**		**Cirrhosis + HCC**	
	
	**Mean ± S.D.**	**median**	**Mean ± S.D.**	**median**
**AFP**	5.96 ± 2.65	5.00	5934.66 ± 13025.02	92.50
**PIVKAII**	23.47 ± 59.69	7.83	135.17 ± 168.96	42.75
**GP3**	125.41 ± 281.05	29.62	161.41 ± 422.33	56.57
**Follistatin**	72.41 ± 76.16	50.20	87.33 ± 131.31	61.35

The mean level between cirrhotic patients versus cirrhotic patients with cancer was significantly different in the cases of AFP and PIVKAII (p = 0.004 and p < 0.001 respectively, Wilcoxon). There was no significant difference between levels for GP3, CD5L or Follistatin.

Mean PIVKA-II levels were also significantly different between patients with HCC and liver cirrhosis, as shown in 1B and Table 2. The median value in the former was 42.74 ng/ml and in the latter 7.8 ng/ml (interquartile range: 2.8 – 17.8). The area under the ROC curve was 0.81 (CI95% 0.715 – 0.886) with a cut-off 20.24 ng/ml. This predicted a sensitivity of 79.6%. (CI95% 65.7% – 89.7%) and a specificity of 80.5% (CI95% 65.1 – 91.2%). As shown in Figure 1F, the level of PIVKAII is significantly raised in patients with tumours >5 cm in size (n = 24) relative to those 3–5 cm in size (n = 23) (ANOVA p = 0.001). In fact, the level from tumours 3–5 cm in size was not significantly different from the level in patients with cirrhosis alone. While there was similarly a difference between these two size groups using AFP (7369 +/- 14361 ng/ml; n = 36 versus 2417 +/- 8889 ng/ml; n = 13), the difference was not statistically significant. Serum AFP therefore is better at specifically detecting early malignant disease than PIVKAII. However, the combination of serum AFP and PIVKA-II in these patients is better than either alone, with a combined sensitivity of 94% and a specificity of just over 80%, as shown in Table 3.

**Table 3:** Performance of combinations of candidate biomarkers

**Combination**	**TP**	**FP**	**TN**	**FN**	**sensitivity (HCC = 50)**	**specificity (LC = 41)**	**TPP**	**TPN**	**LR**
**afp**	28	0	41	22	56.00%	100.00%	100.0%	65.10%	-
**afp+piv**	47	8	33	3	94.00%	80.50%	85.50%	91.70%	4.82
**piv**	39	8	33	11	78.00%	80.50%	83.00%	75.00%	4
**afp+scca**	36	11	30	14	72.00%	73.20%	76.60%	68.20%	2.68
**afp+piv+scca**	45	16	25	5	90.00%	61.00%	73.80%	83.30%	2.31
**piv+scca**	42	16	25	8	84.00%	61.00%	72.40%	75.80%	2.15
**gcp+scca**	36	16	25	14	72.00%	61.00%	69.20%	64.10%	1.85
**afp+gcp**	41	22	19	9	82.00%	46.30%	65.10%	67.90%	1.53
**afp+piv+gcp**	48	27	14	2	96.00%	34.10%	64.00%	87.50%	1.46
**gcp+piv**	46	27	14	4	92.00%	34.10%	63.00%	77.80%	1.4
**all**	48	31	10	2	96.00%	24.40%	60.80%	83.30%	1.27
**gcp**	34	22	19	16	68.00%	46.30%	60.70%	54.30%	1.27
**gcp+piv+scca**	47	31	10	3	94.00%	24.40%	60.30%	76.90%	1.24
**afp+gcp+scca**	36	28	13	14	72.00%	31.70%	56.30%	48.10%	1.05
**scca**	9	11	30	41	18.00%	73.20%	45.00%	42.30%	0.67

The true and false positive (TP and FP), as well as true and false negative (TN and FN) for each candidate either alone or in combination is presented, along with sensitivity, specificity, true percentage positive (TPP) and true percentage negative (TPN) values and the liklihood ratio (LR). The LR is the ratio of true and false positives (sensitivity and 1-specificity respectively), where higher values reflect the probability of a better performance.

### Serum GP3 and SCCA-1 have no role in HCC surveillance in steatohepatitis-related cirrhosis

The data for GP3 and SCCA-1 in this group of ALD/NAFLD patients with and without HCC is also presented in Figures 1 and 2 and Table 2 and 3. These data demonstrate that neither had any value in HCC detection in this group of patients. While their expression was elevated in the serum of patients with chronically diseased livers and HCC, there was no significant difference between the levels detected in cirrhotic patients with and without a cancer.

### Follistatin is raised in the serum of individuals with cirrhosis and HCC, but its specificity for HCC is poor

**Figure 3:**
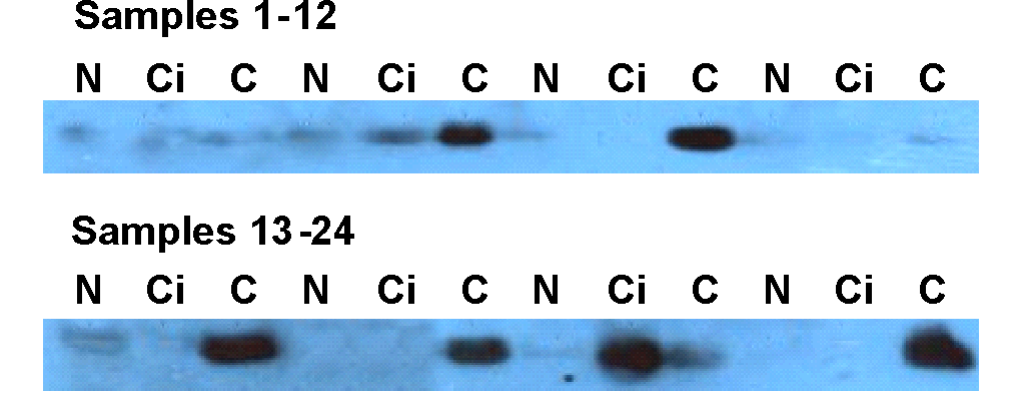
**Follistatin is detectable in serum in patients with NAFLD related HCC**. Immune depleted serum samples from individuals with non-fibrotic NAFLD (N), NAFLD cirrhosis (Ci), and NAFLD cirrhosis with cancer (C) have been separated by SDS-PAGE and analysed by western blot. The majority of HCC patients had detectable levels of follistatin in their serum, as did one or two individuals with NAFLD cirrhosis and no cancer.

Levels of follistatin were studied in immune depleted serum from individuals with either NAFLD (n = 10), NAFLD with cirrhosis (n = 10), or NAFLD with cirrhosis and HCC (n = 10) by western blot analysis. Representative data from 24 of these individuals is presented in Figure 3. While there was little evidence of follistatin in the serum of individuals with NAFLD without significant fibrosis or HCC, it was detectable in all indiduals with HCC as well as some individuals with cirrhosis and no HCC. We went on to develop an ELISA assay for more quantitative raw serum analysis between the latter two groups. Unfortunately, while follistatin is clearly increased in individuals with cirrhotic NAFLD, it fails to distinguish between those with and without HCC, as shown in Figures 1E and 2E.

## Discussion

The increasing incidence of HCC[3], compounded by the fact that the majority of these tumours are diagnosed at a late stage when curative treatments are not possible[19], has prompted the international community into performing regular surveillance of high risk individuals. Unfortunately, surveillance programmes are hindered by the poor performance of the commonly used serum marker, namely AFP[6], even in combination with abdominal USS. A tremendous amount of effort has been and continues to be applied to the search for improved HCC biomarkers. As yet, none has proved superior to AFP in performance, but in combination some may have complimentary roles in HCC arising on a background of viral hepatitis [20]. Our own particular concern relates to the marked increase in the prevalence of ALD and NAFLD related HCC on our own unit.

In our study, serum AFP performs moderately well as a biomarker of HCC in ALD/NAFLD patients, with a sensitivity of 58% (15 ng/ml) in combination with a specificity of 100%. The AASLD recommended cut off level for diagnosis of HCC is 200 ng/ml [21], although lower levels, particularly if rising, should be deemed suspicious and followed very carefully. In ALD/NAFLD patients, where a mild to moderately elevated but stable AFP level similar to that occasionally observed in individuals with viral hepatitis is rare, it may be possible to attach a more sinister connotation to much lower levels of expression. While this data is encouraging, the sensitivity of AFP is not good enough for it to be used in isolation, as over a third of cancers will be missed.

Both the sensitivity and specificity for PIVKAII as an HCC biomarker were in the order of 80% at a level of 20 ng/ml. The addition of PIVKAII serum analysis to that of AFP increases the combined sensitivity to 94%. While this is at the modest expense of the specificity (reduced to 80.5%), the combination of both AFP and PIVKAII analyses may well be justified in our patients. It should be noted, however, that the added benefit is only in the detection of more advanced disease – as indicated in previous viral hepatitis studies and confirmed in our own NAFLD/ALD patients -the encouraging performance of PIVKA-II is predominantly a result of detection of larger, more advanced cancers.

Assessment of the other candidate biomarkers was disappointing. Both the sensitivity and specificity of GP3 were poor in our patient set, indicating that it has no role at all in the surveillance of HCC in individuals with steatohepatitis related cirrhosis. Follistatin is an expressed transcript in fetal liver and has previously been identified by microarray as an up-regulated gene in HCC relative to dysplastic nodules [22]. Although we had high hopes for this activin antagonising protein, [23] based on both our preliminary microarray data and a pilot study in immune depleted sera, the ELISA data assessing its discriminatory function between cirrhotic individuals with and without HCC was poor. The discrepancy between the western and ELISA data is most likely a result simply of assessing a greater number of patients using the latter method, but it is also that follistatin, or an isoform of it, was enriched during the column preparation phase of the serum of HCC patients assessed by western blotting. Perhaps the most surprising of results, however, in this homogenous group of patients with steatohepatitis related HCC, was the disappointing performance of SCCA-1. SCCA-1 has previously shown promise, particularly as an AFP complementary biomarker, in viral hepatitis related HCC [24,25]. In our study in NAFLD/ALD, however, there was no significant difference between levels in patients with and without HCC and while the combination with AFP does modestly improve its sensitivity (78% from 56%), this is at an unacceptable cost to specificity (73% from 100%). Why this serum protein should be significantly elevated in the serum of HCV related HCC patients relative to HCV cirrhosis alone, and not similarly elevated in steatohepatitis related HCC patients relative to steatohepatitis cirrhosis alone is unclear. It is possible that the study of these novel candidate biomarkers complexed to immunoglobulins, rather than the study of their free forms, may yet improve their performance, as has been shown for other biomarkers [26]. Whether or not there is room for improvement, however, our SCCA-1 data clearly indicate that as we come to consider further candidate biomarkers it is important to assess HCC arising in different disease backgrounds independently when performing validation studies.

## Conclusion

In summary, while we propose the combination of AFP and PIVKAII for HCC surveillance in NAFLD/ALD patients, the search for novel biomarkers of early HCC disease should continue.

## Competing interests

The authors declare that they have no competing interests.

## Authors' contributions

DC has collected samples and with supervision performed the western blotting and the majority of ELISA assays, GB has directly supervised the ELISA assay optimisation and data collection, JG has optimised the method of serum preparation for subsequent analysis, SS, MH, CD have contributed to the study design and recruited patients to the study, PT has contributed to the study design and performed much of the statistical analysis, GG has performed all of the SSC1 biomarker ELISA assays and contributed substantially to data analysis and manuscript preparation, DM and HR conceived the study, contributed to its design and co-ordination, HR has completed final data analyses and written the manuscript. All authors read and approved the final manuscript.

## Pre-publication history

The pre-publication history for this paper can be accessed here:


